# Nanoplastics released from daily used silicone and latex products during mechanical breakdown

**DOI:** 10.1371/journal.pone.0289377

**Published:** 2023-09-13

**Authors:** Mikael T. Ekvall, Isabella Gimskog, Egle Kelpsiene, Alice Mellring, Alma Månsson, Martin Lundqvist, Tommy Cedervall

**Affiliations:** 1 Aquatic Ecology, Lund University, Lund, Sweden; 2 NanoLund, Lund University, Lund, Sweden; 3 Biochemistry and Structural, Biology, Lund University, Lund, Sweden; King Abdulaziz University, SAUDI ARABIA

## Abstract

Waste of polymer products, especially plastics, in nature has become a problem that caught the awareness of the general public during the last decade. The macro- and micro polymers in nature will be broken down by naturally occurring events such as mechanical wear and ultra-violet (UV) radiation which will result in the generation of polymeric particles in the nano-size range. We have recently shown that polystyrene and high-density polyethylene macroplastic can be broken down into nano-sized particles by applying mechanical force from an immersion blender. In this article, we show that particles in the nano-size range are released from silicone and latex pacifiers after the same treatment. Additionally, boiling the pacifiers prior to the mechanical breakdown process results in an increased number of particles released from the silicone but not the latex pacifier. Particles from the latex pacifier are acutely toxic to the freshwater filter feeding zooplankter *Daphnia magna*.

## Introduction

The problem with polymeric waste, especially plastics, in nature, has been highlighted in the last decade [1–13]. Nanoplastics from broken-down plastic and rubber waste may pose a more severe threat to aquatic wildlife than macropolymers owing to the impact of their small size on their environmental fate, exposure scenarios and biological impact [2, 14, 15]. Size-dependent toxicity has been shown in zooplankton exposed to polystyrene nanoparticles (NPs) [16, 17]. However, most of what we know about the biological effect of nanoplastics derives from studies using commercially manufactured primary polystyrene nanoparticles [17–21]. These particles are well defined in terms of size and surface chemistry and have allowed studies determining the importance of size, different surface chemistry, and for controlled alterations of the particles. However, nanoplastics in nature is expected to substantially differ from commercially obtained nanoplastics [22].

Silicone is a versatile material used in a broad range of products, from flexible baking moulds to flexible and stretchable electronics [23]. However, silicone is not just one material but several, with the common features that silicon atoms are linked via oxygen atoms and each silicone atom bears one or several organic groups [24]. Likewise, latex is a broad concept and is described as “Latex is an example of a colloidal dispersion. It consists of polymeric particles, which are usually a few hundred nanometres in diameter, dispersed in water. The particles typically comprise about 50% by weight of the dispersion. Depending on the application, there will also be a complex mixture of pigments, surfactants, plasticising aids and rheological modifiers. The whole dispersion is colloidal stable, meaning that it can sit on a shelf for years and remain dispersed, without sedimentation of particles making “sludge” at the bottom” [25].

We recently published articles in which we presented a protocol to mimic the mechanical wear that macroplastics waste may be exposed to in nature, such as rubbing against other waste in the waves or against sand and stones on shorelines. By using an immersion blender for 5 min nanoplastics were released from polystyrene (PS) and high-density polyethylene products (HDPE) [26, 27]. Here we present the results from two pacifiers of silicone and natural rubber latex (NRL), before and after boiling of the pacifiers. Additionally, the acute toxicity of the generated nanoparticles has been evaluated using the freshwater zooplankter *D*. *magna* as a model organism.

## Material and methods

### Particles preparation and characterization

In this study we have focused on two materials, silicone and latex. Pacifiers manufactured by ESSKA, www.esska.nu, with a silicone rubber or latex rubber bubble. The mechanical break-down of plastic items was done according to previously published protocol [26, 27]. In short: the macro plastic, 2 g of material, was added to a 500 mL glass beaker together with 115 mL of MilliQ H_2_O and mixed for five minutes using an immersion blender (BOSCH ErgoMixx 600 W) at its highest rpm. The samples were then filtered through 0.8 μm syringe filters, Whatman FP 30 / 0.8 CA Filter Unit, before any analysis or test were conducted.

Three techniques were used to characterize the particle size distribution of the produced samples, Nanoparticle tracking analysis (NTA), dynamic light scattering (DLS) and transmission electron microscopy (TEM).

### Boiling treatment of the pacifiers

The silicone and latex pacifiers were boiled in 100 mL of MilliQ H_2_O for 10 min and allowed to cool down to room temperature. A 1 mL sample was withdrawn, and MilliQ H_2_O was added, to compensate for evaporation, so the volume once again was 100 mL and the procedure were repeated 7 times for the silicone pacifier and 3 times for the Latex pacifier.

### Nanoparticle tracking analysis

NTA measurements were performed using a NanoSight LM10 instrument (Nanosight, England) equipped with a 405 nm laser. Videos were recorded for 60 s using the same camera level (14) for both particle and control (pure MilliQ H_2_O) samples and analyzed by using NTA 2.3 software (Nanosight, England) or NTA 3.1 software (Nanosight, England). The size data are reported as the mean (by concentration) and mode values.

### Dynamic light scattering & zeta potential

Size and zeta potential measurements were performed using a Zetasizer Nano ZS instrument using DTS1070 cuvettes (Malvern Instruments, Worcestershire, UK). The samples were equilibrated at 25°C before the start of the measurement. Each sample were measured three times and the data is represented as mean values ± standard deviation.

### Transmission electron microscopy

TEM was performed using a JEOL JEM-1400 PLUS microscope equipped with a JEOL Matataki CMOS camera at 100 kV (JEOL Ltd., Japan). The samples were prepared by pipetting 2 μl of sample onto a pioloform-coated single slot grid (Ted Pella, Cu, Pelco Slot Grids, USA). The water was let to evaporate from the grid before imaging. The images were taken using the JEM1400 Plus software.

### Fourier-transform infrared spectroscopy

Attenuated total reflectance Fourier transformed infrared spectrometry (ATR-FTIR) was performed on a Spectrum One FTIR spectrometer (Perkin Elmer) equipped with a Universal ATR accessory. 2 x 5 μl, for a total volume of 10 μl, of each sample were added to the ATR-crystal and allowed to completely dry (the ATR-crystal was dry before the second 5 μl of the sample was added) before the measurement. The lever was used to ensure good contact with the crystal. The spectra were obtained using software Spectrum (version 6.2.0), a Spectral resolution of 4 cm^-1^, a range of 4000–550 nm and 50 scans per sample.

### Acute toxicity

Acute toxicity tests on the produced breakdown particles (see above) were performed using the freshwater zooplankter *D*. *magna*. One individual was put in each tube with 20 mL either breakdown particle solution (silicone or latex treatments) or tap water only (control). The particle concentration was approximately 2 x 10^7^ particles/mL. There were 15 replicates for each treatment. The experiment was repeated twice. All experimental groups were kept at 18 ֯C temperature with 12:12 hours light:dark cycle. The experiment lasted for 72 hours, and the *Daphnia* were not fed during the experiment. Survival was recorded every 24 hours. Survival was evaluated using Kaplan-Meier survival analysis using GraphPad Prism (version 8.4.3) and the p-value was attained from the log-rank (Mantel-Cox) test.

## Results and discussion

We established the protocol for mechanical breaking down macroplastic into a mixture of nano-, micro- and macroplastic in 2019 [26]. We want to stress the importance of control samples in these types of experiments. We were very lucky when we bought the blender in 2018 as a small investigation of different kitchen blenders revealed that they all released various amount of material in the nano size range in control experiments see SI material, S1-S4 Figs and S1 Table in S1 File.

**Fig 1:**
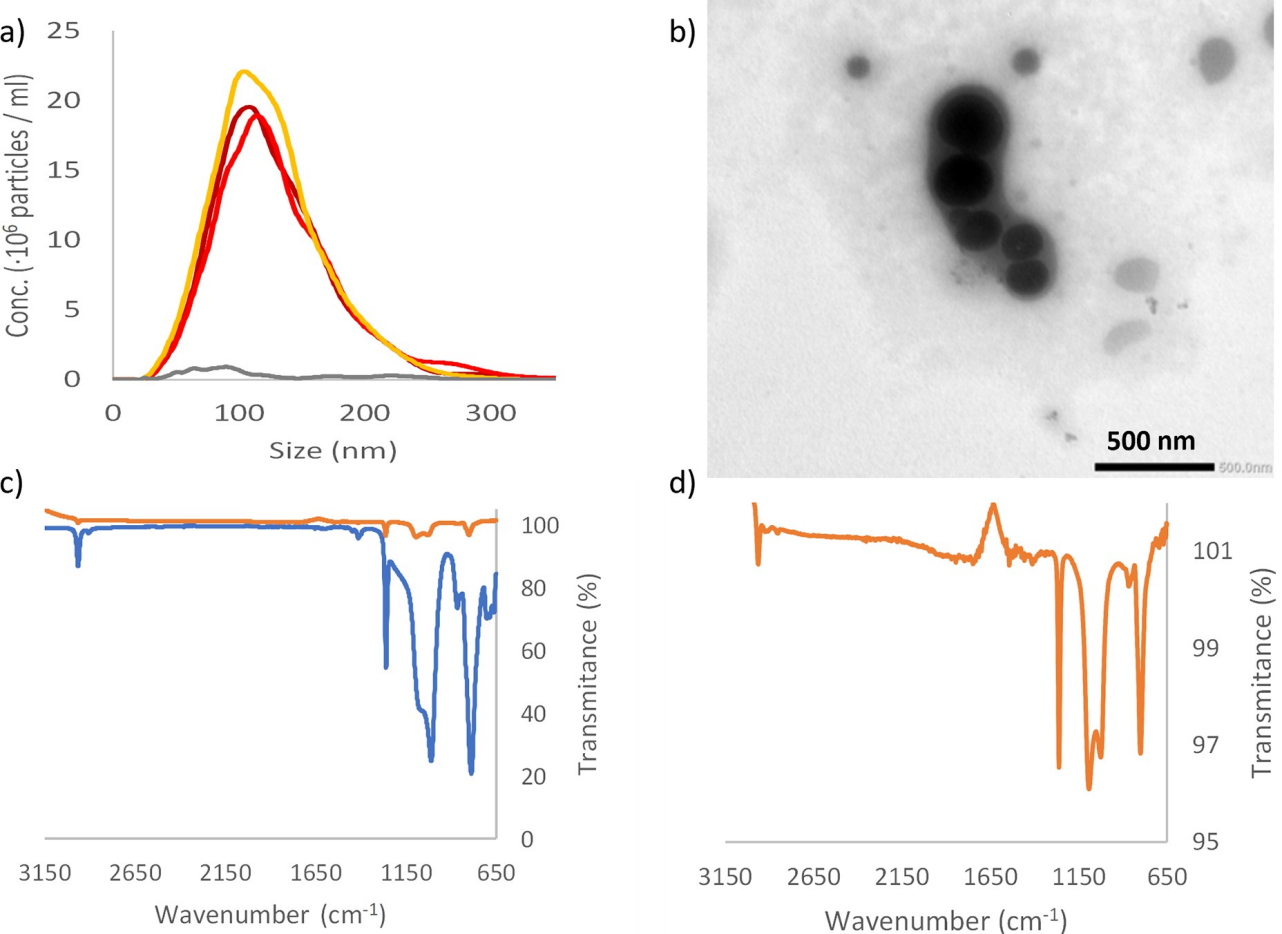
Characterization of breakdown product from silicone pacifier. a) NTA data from three repeats of breakdown of silicone pacifier (dark red, red and orange lines) and control, blended water (grey line). b) TEM picture of the produced silicone nanoparticles. c) ATR-FTIR data of the material before breakdown (blue line) and after breakdown and lyophilized (orange line). d) show a zoom in on the lyophilized breakdown data.

The size distribution measured by NTA, for three separate samples of silicone pacifier broken down in MilliQ H_2_O is shown in Fig 1A. It is evident that NPs are formed after 5 min mechanical stress. The mean sizes of the NPs is 129 nm and 182 nm measured by NTA and DLS, respectively, whereas the most common particle size (mode) in the NTA measurements is smaller, see Table 1 and Fig 1. The size distribution of the formed nanoparticles is narrow, Fig 1A, and surprisingly there are almost no particles detected with a size over 300 nm by NTA. Fig 1C shows the ATR-FTIR data for the starting material and the produced NPs. The spectra, blue in Fig 1C), shows peaks around 2963 cm^-1^ (methyl CH), 1258 cm^-1^ (Si-CH_3_), 1080 and 1008 cm^-1^ (Si-O-Si), and 784 cm^-1^ (Si-C) and/or (Si-(CH_3_)_2_) well coincide with those reported in the literature [28–31]. The spectra of the formed NPs, dark red in Fig 1C and 1D, are almost identical with the bulk material, with peaks around 2963 cm^-1^, 1261 cm^-1^, 1092 cm^-1^, 1022 cm^-1^, and 799 cm^-1^. The ATR-FTIR data shows that the detected NPs are silicone based and that the breakdown of the bulk material into nanoparticles does not significantly affect the chemistry of the silicone.

**Table 1:** Characterization of nanoparticles produced by using immersion blender.

	NTA		DLS		Z-potential
Sample	Mode (nm)	Mean (nm) ±SD	Diameter (nm) ±SD	PDI	(mV) ±SD
Silicone pacifier	109	129 ± 47	182 ± 2	0.41	-16.9 ± 0.3
Latex pacifier	105	143 ± 59	742 ± 21	0.57	-6.2 ± 0.4

**Fig 2:**
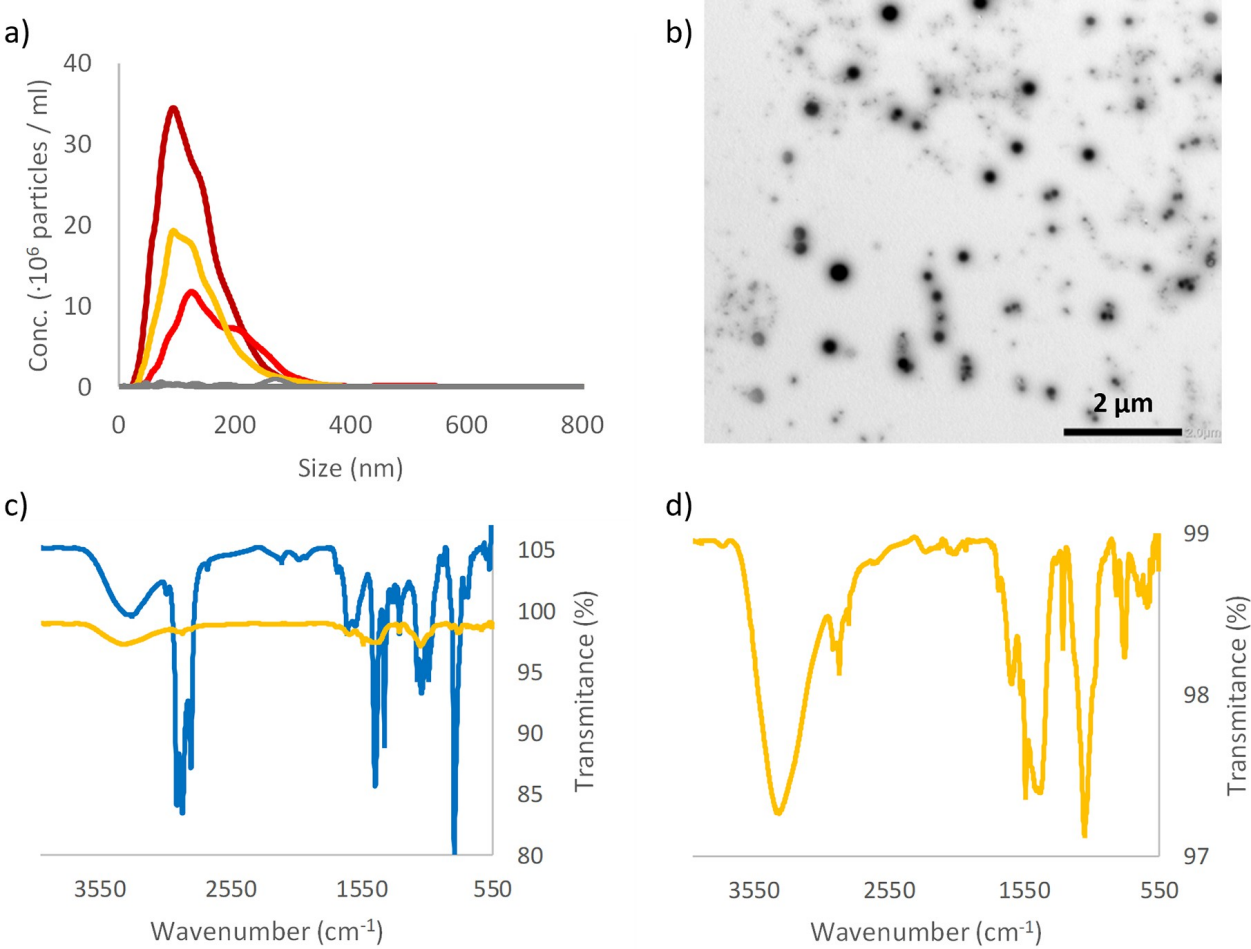
Characterization of breakdown product from latex pacifier. a) NTA data from three repeats of breakdown of latex (dark red, red and orange lines) and control, blended water (grey line). b) TEM picture of the produced silica nanoparticles. c) ATR-FTIR data for the material before breakdown (blue line) and after breakdown and lyophilized (orange line). d) show a zoom in on the lyophilized breakdown data.

As stated in the introduction, latex is a very complex mixture. Therefore, we first used ATR-FTIR to identify the material. The FTIR spectrum obtained, see Fig 2C, is similar to other published spectra for NRL [32–36]. NRL, from rubber tree *Hevea brasukuensis*, is a colloidal system composed by 30–45% rubber particles, and 4–5% non-rubber constituents (such as proteins, lipids, and carbohydrates) and with water accounting for the remaining percentage [37]. The FTIR spectra recorded for latex in this article shows a close resemblance to the FTIR spectra published by Pongsathit et al. with extra absorbance peaks between wavenumber 1000–1400 cm^-1^ [33], hence, we assume that the material tested in this article is, similar to, NRL.

After mechanical breakdown of the latex pacifier NPs are produced with mean sizes of 143 nm and 742 nm according to NTA and DLS, respectively, Fig 2A and Table 1. As for the silicone pacifier the most common size is smaller than 150 nm according to NTA. However, in difference from the silicone pacifier and other materials tested, PS [26], and HDPE [27] the number of particles produced, and the size distribution varies more when the breakdown is repeated, Fig 2A. TEM image (Fig 2B) reveals that the produced sample contains a mixture of differently sized particles after mechanical breakdown of Latex. The ATR-FTIR spectra for the produced particles have absorbance peaks at 2960 cm^-1^, 2916 cm^-1^ and ~1452 cm^-1^ that also was found in the bulk material, Fig 2C and 2D. These peaks correspond to -CH_3_ and -CH_2_- stretching and C-H bending of CH_2_ respectively. In addition, the FTIR spectra for the produced NPs show new absorbance peaks, mainly in the region 550–1750 cm^-1^, i.e., the chemistry is clearly altered when the bulk material is disintegrated into NPs. Kim et al. have shown the ATR-FTIR spectra of natural rubber thin film change after UV oxidation [36] and we speculate that similar oxidative processes can occur when we degrade the material into nanoparticles, i.e. the significantly increase of the surface area / material mass ratio would help the process. Contrariwise to Kim et al. study, the bulk material used in this study already shows a broad absorption peak at 3100–3600 cm^-1^ corresponding to hydroxyl and a peak at 1734 cm^-1^ corresponding to carbonyl. The FTIR spectra from the formed NPS show less absorbance at the carbonyl wavenumber compared to the hydroxyl absorbance compared to the bulk material which would rule out oxidation. However, since the FTIR spectra of the formed NPs our recorded after the particles, in a colloidal solution in water, had dried to form a layer on the ATR-crystal it is possible that a part of the observed absorption peak at 3100–3600 cm^-1^ derives from water molecules associated with the huge surface area of the NPs.

The higher degree of oxidation of the breakdown NPs observed for the latex has also been observed for the two plastics, PS and HDPE, that we have previously investigated [26, 27]. In contrast, no oxidation is observed on the silicone NPs, indicating that this material is more stable after the mechanical breakdown. This may indicate that carbon-based breakdown particles undergo chemical changes while silicone-based breakdown particles do not. There are at least two interesting possibilities arising from this observation. One is that the breakdown of silicone-based rubber will be slower compared to carbon-based rubber and plastics. The other is that the possible interaction with elements in nature will not change to the same degree when silicone rubber is breaking down.

**Fig 3:**
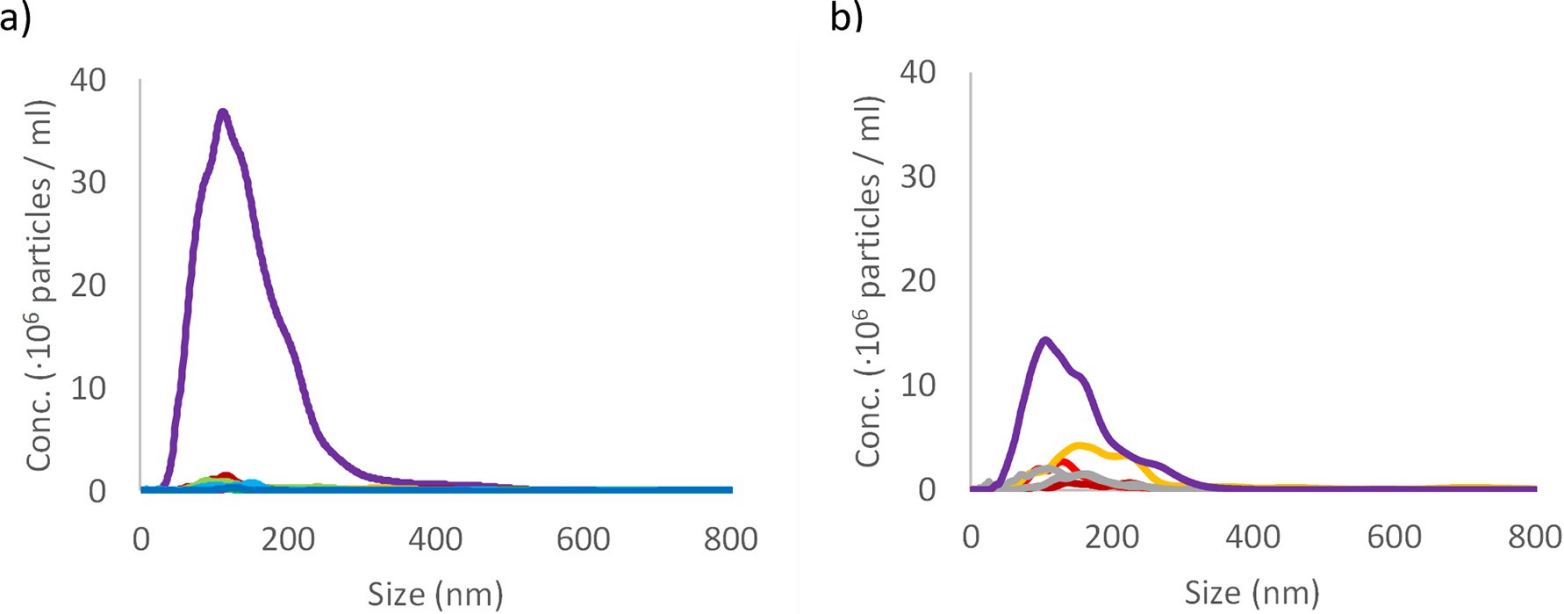
Particle size determination after boiling pacifiers. NTA data for boiled samples. Panel a) particle count data from seven repeats of boiling of silicone pacifiers in different colours and after mechanical breakdown of the boiled material in purple. Panel b) data from three repeats of boiling of latex in dark red, red and orange lines and after mechanical breakdown of the boiled material in purple line. Two control samples are shown in grey lines.

As pacifiers are often sterilized by boiling before use, the silicone and latex pacifier were subjected to heat treatment to evaluate if repeating heating of the material would generate a detectable number of NPs. The boiling of the material does not in itself generate a detectable number of NPs, Fig 3. This is interesting as heat treatment of nylon tea bags has been shown to release a large number of micro- (∼200 μm) and nanoplastics (between ∼100 nm and ∼1000 nm) [38]. Next, we wanted to test if the boiling treatment of silicone and latex pacifiers affects the release of NPs after mechanical breakdown. The heat-treated silicone pacifier release approximately twice the number of NPs whereas the release from heat treated latex is the same as for untreated, Fig 3, and compare with Figs 1A and 2A.

The possible toxicity of the silicone and latex breakdown NPs was tested on *D*. *magna*. All particle solutions and control group were prepared in tap water. S2 Table in S1 File reports NTA data for silicone and Latex pacifiers after being subjected to 5 min mechanical wear in tap water and S5 and S6 Figs in S1 File shows the corresponding NTA data. The size distribution and the released number of NPs are similar to the breakdown experiments performed in MilliQ water.

**Fig 4:**
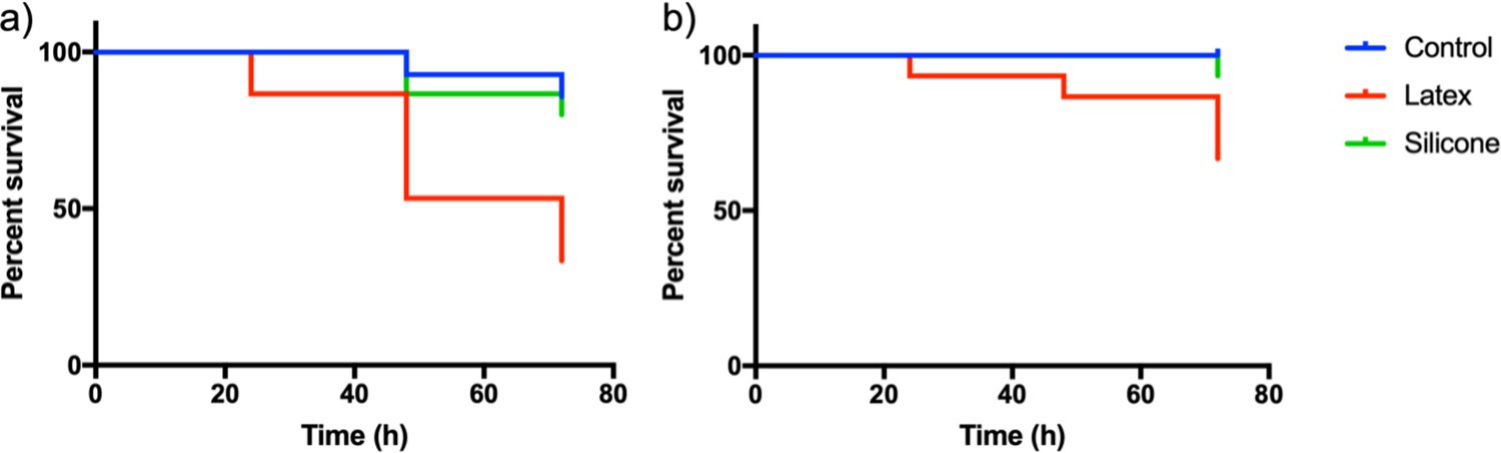
Kaplan-Meier survival curves. Kaplan-Meier survival curves for two consecutive acute (72 h) toxicity tests on *D*. *magna* in presence of breakdown NPs derived latex and silicone. Both graphs show a significant decrease in survival for *D*. *magna* in the breakdown latex samples.

The survival of *D*. *magna* in latex breakdown NPs is significantly lower in both tests compared to the control in both the first (χ^2^_(df = 1)_ = 9.091, p = 0.0026, Fig 4A) and second exposure test (χ^2^_(df = 1)_ = 5.785, p = 0.0162, Fig 4B). This is perhaps not surprising as latex stoppers, material on macro scale, in contact with water has already in 1965 been shown to be toxic to *D*. *magna* [39]. In contrast the silicone NPs do not affect the survival compared to the control (χ^2^_(df = 1)_ = 0.2447, p = 0.6208, Fig 4A, and χ^2^_(df = 1)_ = 1.0, p = 0.3173, Fig 4B). The particle concentration is approximately 2 x 10^7^ particles/mL which roughly corresponds to 20 and 50 μg/L for latex and silicone, respectively. The concentration of latex and silicone NPs in nature is not known but the mean concentration of nanoplastics has been reported to be 563 μg/L in rural Swedish lakes [40].

During the two acute toxicity tests, it was observed that *D*. *magna* individuals together with latex NPs got stuck to the water/air interface, see S7 Fig in S1 File. S7 Fig in S1 File shows a microscope image of a dead *Daphnia* from the latex group compared to one from the control group. We currently do not have a certain explanation for these observations. However, one possible explanation could be that the carapace of the *D*. *magna* has become hydrophobic from the latex-particles attaching to it, and thereby interacting with the air phase.

## Conclusions

These result shows that both silicone and latex pacifiers released nanoparticles when subjected to mechanical wear. Boiling of the silicone or NRL samples did not by itself make the macro material release a significant number of nanoparticles. However, when the boiled silicone pacifier was mechanically broken down, a significant increase of the number of produced nanoparticles could be observed. Acute (72 h) toxicity test with *D*. *magna* revealed that the latex NPs, but not the silicone NPs, show significant toxicity. The mechanisms for the observed toxicity in the latex treatment may be related to that the NPs causing the carapace of the *D*. *magna* to become hydrophobic. All the particles produced from the silicone material was shown to be more benign in terms of toxicity as compared to the produced latex particles.

## Supporting information

S1 File (PDF)

S1 Data (XLSX)
