# Supplementary material for: Nanoplastics released from daily used silicone and latex products during mechanical breakdown
Source: PLoS One. 2023 Sep 13;18(9):e0289377. doi: 10.1371/journal.pone.0289377 (PMC10499202; doi:10.1371/journal.pone.0289377)
Supplement: S1 File — (PDF) [file pone.0289377.s001.pdf]

Supporting information

Nanoplastics released from daily used silicone and latex products during mechanical breakdown

Mikael T. Ekvall[1, 2*], Isabella Gimskog[3], Egle Kelpsiene[2, 3], Alice Mellring[3], Alma Månsson[3], Martin Lundqvist[2, 3] and Tommy Cedervall[2, 3]

Material

Silicone

Silicone 1 was bought from www.apotea.se, and were pacifiers manufactured by ESSKA, www.esska.nu, with a silicone rubber bubble. Silicone 2 was from illbruck, www.illbruck.com. illbruck GS202 is silicone used in building constructions as a sealing material.

Latex

Latex was bought from www.apotea.se, and were pacifiers manufactured by ESSKA, www.esska.nu, with a latex rubber bubble.

Immersion blender

In order to validate how robust, the immersion blender protocol is when it comes to produce wear NPs from plastics, four different immersion blenders were tested: Electrolux ESTM 500, BOSCH powermaxx 750 W, Robot coupe Mini MP 240 v.v. and BOSCH ErgoMixx 600 W, see Figure S1.

The test was set up by blending MilliQ H_2O in a 500 mL glass beaker at their maximum speed for 5 min. Of the tested blenders, it was only BOSCH ErgoMixx 600 W that did not release a significant number of particles in the nano size range (see Figure S2 and S3).

The results for BOSCH powermaxx 750 W can be seen in Figure S3, since it released significant more NPs compare to the other mixers. The mixer was tested at two different times. The data reported here is the data from the second test in which the samples, three repeats, were diluted 10 times with MilliQ H_2O in order to get a concentration that work for the NTA. The data for the BOSCH powermaxx 750 W were collected and analysed using the software NanoSight NTA version 3.1.
The NTA data for the control experiments are summarized in Table S1.

Problems with BOSCH ErgoMixx 600 W

We have so far bought four complete (both motor and knife) sets of BOSCH ErgoMixx 600 W. Three of these have worked, in the sense that they did not release nanoparticles in control experiments. We did also buy ten separate new knives from the manufacture, however, all

of them were useless for our purpose. To our surprise, all of them released material in the control experiment, some of them released something that actually miss-coloured the water, see Figure S4. Our interpretation of these findings is that the knife probably has a shelf life, hence, if it is too old some component in it will start to release material during use.

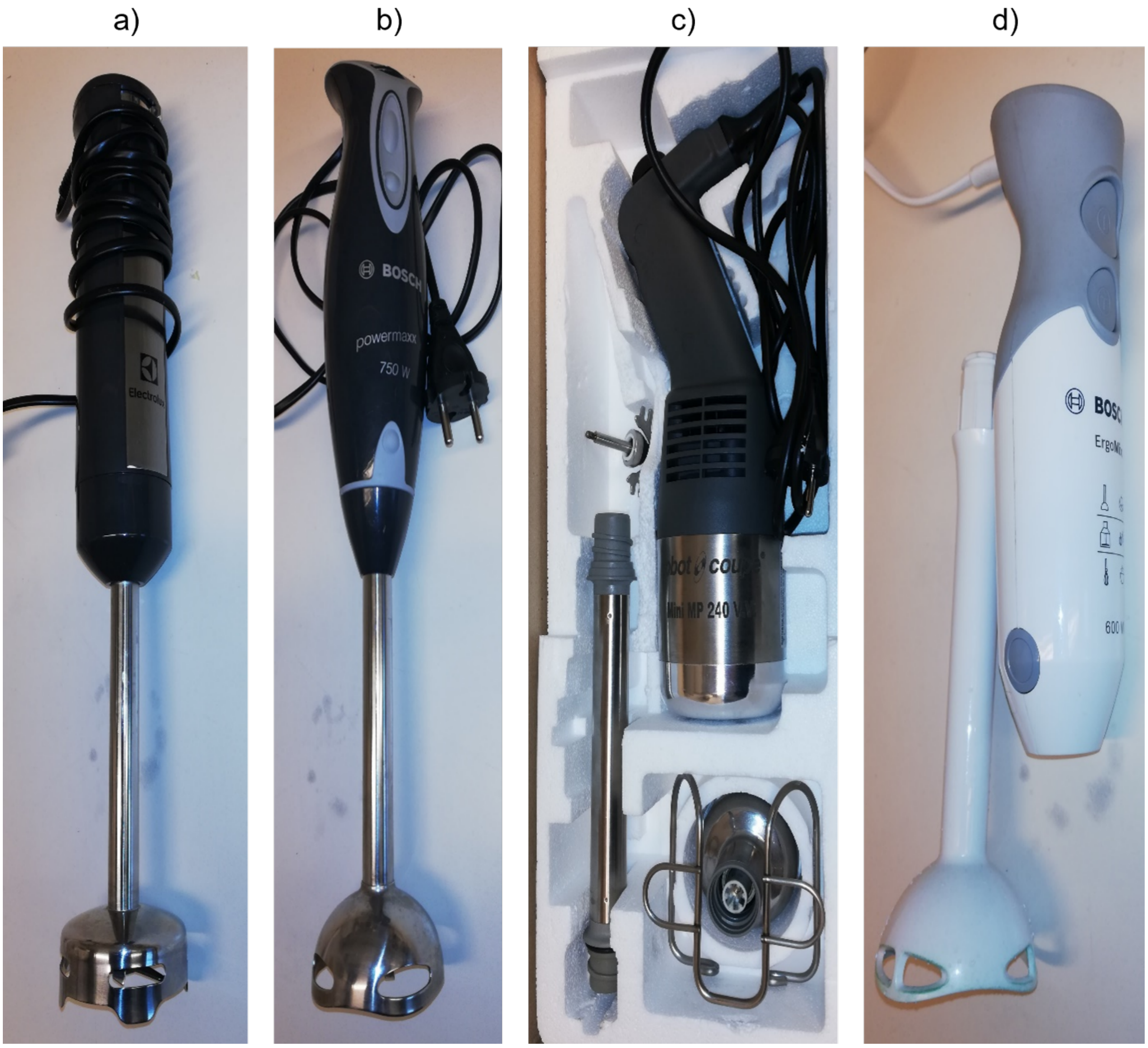

Fig S1. Different immersion blenders tested. a) Electrolux ESTM 500 b) BOSCH powermaxx 750 W c) Robot coupe Mini MP 240 v.v. and d) BOSCH ErgoMixx 600 W.

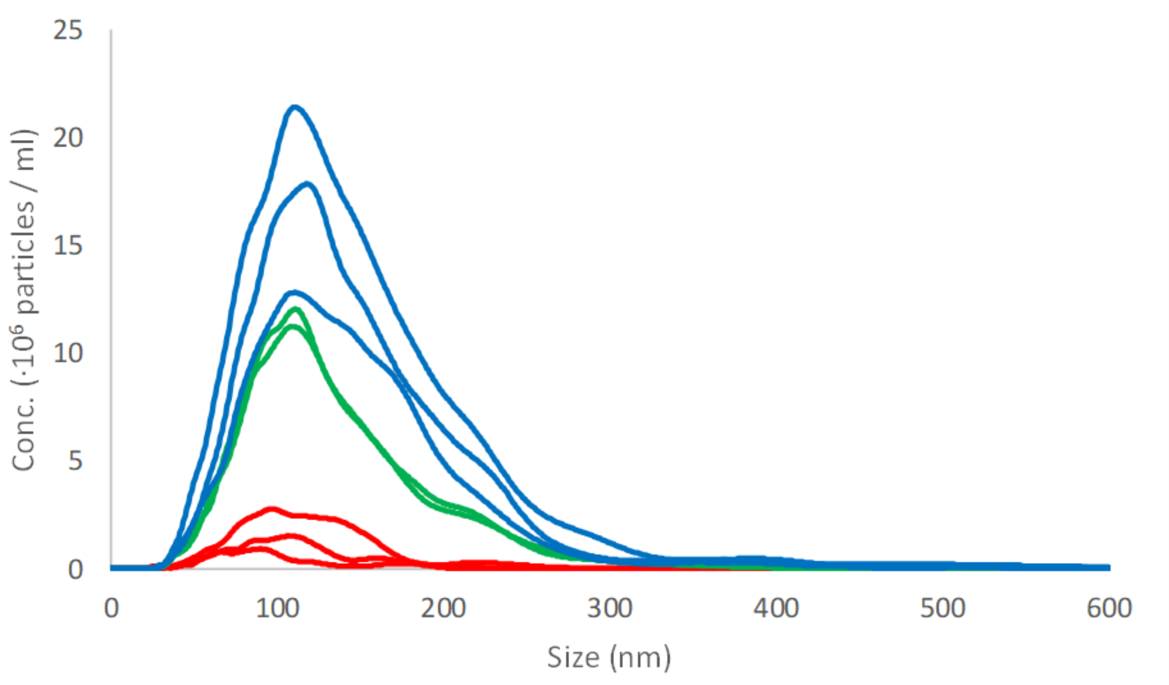

Fig S2. NTA data of control experiments for different immersion blenders. Different immersion blenders particle release after 5 minutes mixing of water. Green = Electrolux ESTM 500, Blue = Robot coupe Mini MP 240 v.v. and Red = BOSCH ErgoMixx 600 W. Run with Camera Level 13.

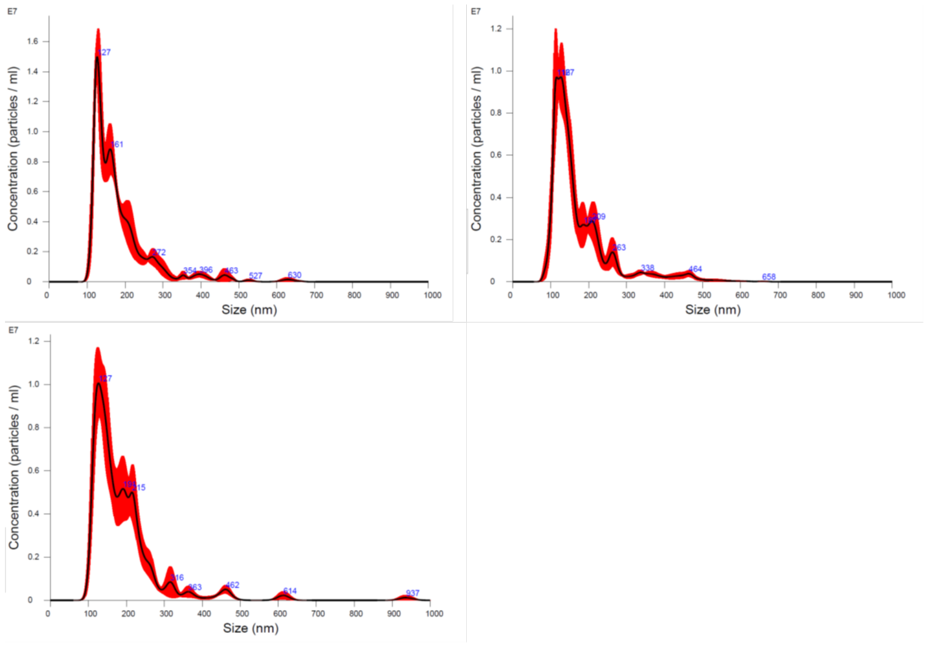

Fig S3. NTA measurements on the samples for the BOSCH powermaxx 750 W immersion blender. Each graph shows the medium value, black line, and the standard deviation, red area, for 5 separate measurements of three different preparations with the mixer. The samples were diluted 10 times with MilliQ water before the NTA measurement.

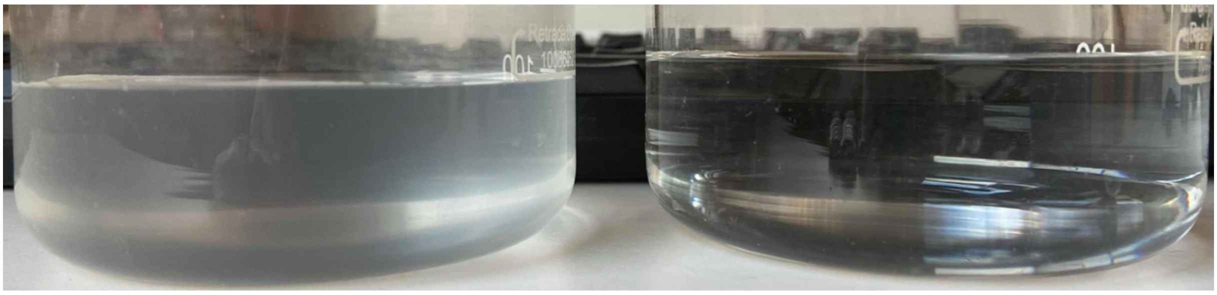

Fig S4. Picture of water after using different knives. To the left, MilliQ H_2O mixed 1 min with BOSCH ErgoMixx 600 W replacing knife. To the right control (unmixed MilliQ H_2O).

Table S1. NTA data for tested Immersion Blenders.

Run and analysed with NTA software NanoSight NTA version 2.3	Camera Level	Mode	Mean	Equivalent particle concentration	Valid Tracks
		(nm)	(nm)	($\cdot 10^8$ particles/ml)	(No.)
Electrolux ESTM 500	13	111	151 ± 108	12,3	2582
Electrolux ESTM 500	13	110	140 ± 67	12,1	2460
Robot coupe Mini MP 240 v.v.	13	111	146 ± 64	26,3	4733
Robot coupe Mini MP 240 v.v.	13	111	149 ±71	16,3	2892
Robot coupe Mini MP 240 v.v.	13	118	151 ± 75	20,9	3884
BOSCH ErgoMixx 600 W	13	90	125 ± 67	0,8	158
BOSCH ErgoMixx 600 W	13	97	114 ± 33	2,5	525
BOSCH ErgoMixx 600 W	13	109	111 ± 40	1,2	233
Run and analysed with NTA software NanoSight NTA version 3.1					
BOSCH powermaxx 750 W	12	126	185 ± 82	10,6	N.A.[1]
BOSCH powermaxx 750 W	12	126	191 ± 98	10	N.A.[1]
BOSCH powermaxx 750 W	12	127	175 ± 86	8,2	N.A.[1]

[1] N.A.: Not Applicable since the reported values are the average of five individual measurements.

Table S2. NTA data for silicone and latex pacifiers after being subjected to 5 min mechanical wear in MilliQ respectively tap water.

		NTA	
		Mode (nm)	Mean (nm)
Silicone 1	MilliQ H_2O	109	129 ± 47
	Tap H_2O	108	143 ± 34
Latex	MilliQ H_2O	105	143 ± 59
	Tap H_2O	163	218 ± 131

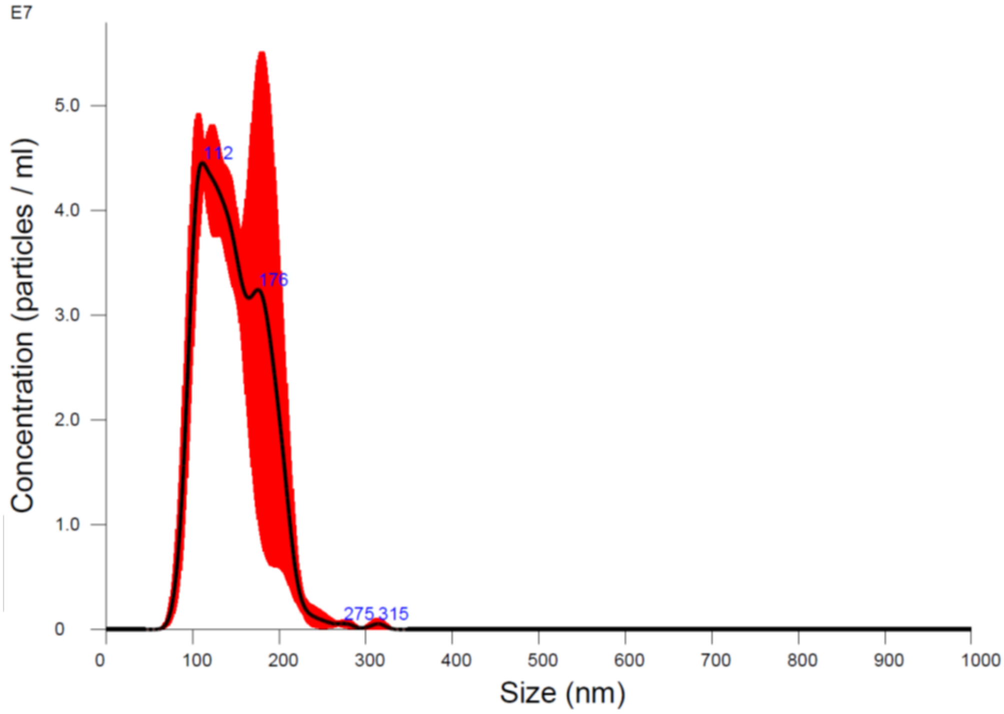

Fig S5. NTA data for Silicone in tap water. The graph shows the medium value, black line, and the standard deviation, red area, for 3 separate measurements of one sample. Data were generated and analysed with NanoSight NTA version 3.1.

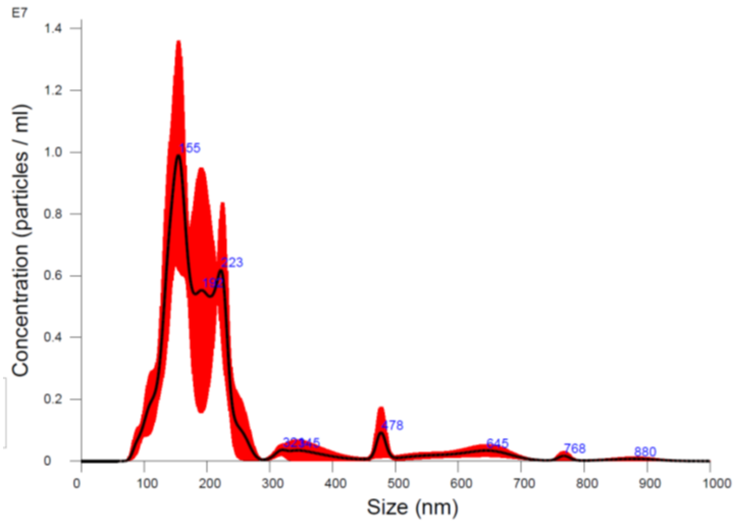

Fig S6. NTA data for Latex in tap water. The graph shows the medium value, black line, and the standard deviation, red area, for 3 separate measurements of one sample. Data were generated and analysed with NanoSight NTA version 3.1.

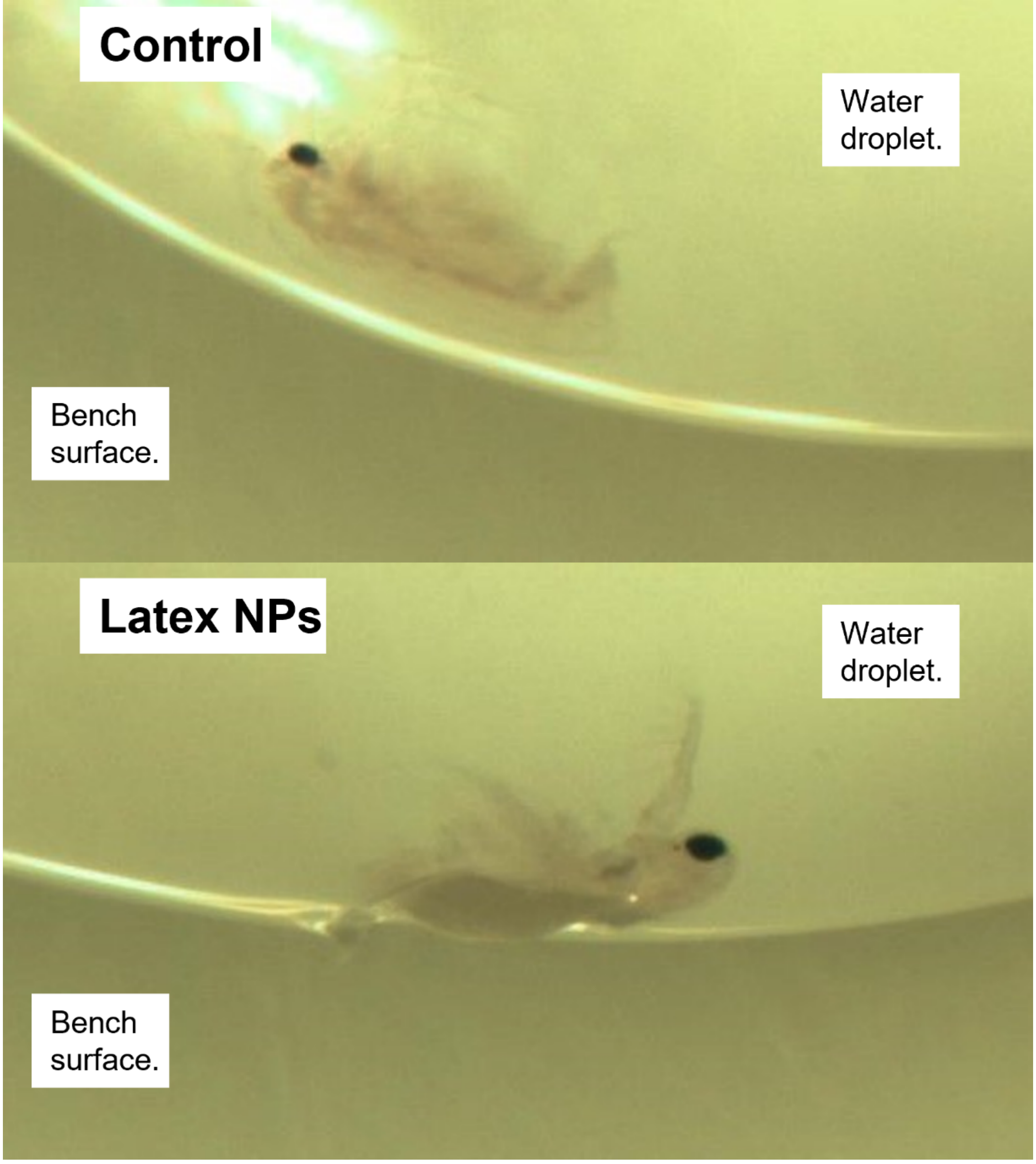

Figure S7. Droplet of control sample with D. magna on the top and droplet of latex sample with D. magna on the bottom.
